# The development of hamstring strength over the course of a simulated soccer match

**DOI:** 10.1371/journal.pone.0315317

**Published:** 2024-12-13

**Authors:** Dominic Michael Rasp, Florian Kurt Paternoster, Michael Zauser, Jan Kern, Ansgar Schwirtz

**Affiliations:** 1 Biomechanics in Sports, Technical University of Munich, Munich, Bavaria, Germany; 2 Human Movement Science, Technical University of Munich, Munich, Bavaria, Germany; Portugal Football School, Portuguese Football Federation, PORTUGAL

## Abstract

Hamstring strain injuries are a prevalent burden in soccer. Low strength, muscle fatigue, and inter-limb differences in hamstring strength are associated with hamstring injury risk. Previous research shows increased hamstring injury incidence in soccer at the end of each half or the end of the match, respectively. This study aims at evaluating the aforementioned risk factors of hamstring injury over the course of a simulated soccer match. Ten active soccer players carried out the Loughborough Intermittent Shuttle Test, during which hamstring strength of both legs was assessed on seven occasions via the optimized 90:20 Isometric Posterior Chain Test. Hamstring strength of each limb and inter-limb differences in hamstring strength over the course of the Loughborough Intermittent Shuttle Test were parameters of interest. Repeated measures ANOVA were used to analyze the development of hamstring strength and limb-asymmetries in hamstring strength during the simulated soccer match. Compared to pre-match values, hamstring strength was significantly decreased after 15 and 30 minutes of simulated soccer match for the non-dominant and dominant leg, respectively. There were no further variations in hamstring strength within the simulated soccer match for either leg. We did neither measure significant recovery of hamstring strength to pre-match values at the beginning of the second half, as suspected by previous research, nor inter-limb differences, or a deterioration of limb asymmetries in hamstring strength during the simulated soccer match. Players who only participate for a short period in a soccer match may be exposed to the same risk of suffering hamstring injury like players who compete for a longer duration. Decreasing hamstring strength partly depicts the pattern of hamstring injury incidences during soccer matches. Additional factors may influence the increasing hamstring injury rate at the end of each half or the later stages of a match, respectively.

## Introduction

Hamstring strain injuries (HSI) are the most frequent type of injury in soccer [1]. The risk of suffering HSI during a soccer match is increased by the 8 to 15-fold compared to training [1–5]. Inter-limb differences in hamstring strength (HSS) [6–10] and low HSS [8, 11–15] are associated with an increased risk of suffering HSI. On top of that, insufficient recovery time between matches [16–19] or after intense bouts of running or sprinting within a match [20] also enhances the risk of HSI. Furthermore, within a soccer match, the risk of suffering HSI has been observed to increase over the course of each half with the last 15 minutes of each half accounting for more HSI than the first 30 minutes [1, 3, 21]; this also accounts for thigh injuries [2], and muscle strains in general [22]. In contrast to that, Cloke et al. [23] recorded thigh injuries in youth soccer–irrespective of muscle–and observed a significant, increasing risk of injury until the second quarter of the match with no further increase in the risk of suffering HSI in the third and fourth quarter. Measuring HSS, or hamstring muscle fatigue, respectively, on several occasions over the course of a real soccer match is not feasible. This is why simulated soccer matches, which mimic the physical demands of a real soccer match by repeated running and sprinting activities on a treadmill or overground, feature an adequate alternative for that purpose. The assessment of HSS on each of the various occasions during a simulated soccer match, however, has to be time-efficient and should not put the players at additional risk of HSI, why objective, reliable and valid isometric tests, such as the optimized version of the 90:20 isometric posterior chain test (90:20 IPCT), are suitable to do so [24].

Numerous studies assessed pre- vs. post-match HSS during a real [25–30] or simulated [31–39] soccer match, or included an additional half-time measurement [40–43]. In contrast to that, only few studies tracked the development of HSS over the course of a (simulated) soccer match on further occasions to gain insight into hamstring fatigue within the match [44–49]. None of these studies [44–49], however, analyzed both legs, which does not allow any conclusion about hamstring muscle fatigue of both the dominant and the non-dominant leg over the course of a soccer match, although both limbs are equally affected by HSI [50]. Furthermore, some research indicates that there is leg dominance in running and change of direction tasks why one leg contributes more force in steady state running, and acceleration and deceleration phases than the other [51–53]. Since limb asymmetries contribute to the risk of HSI, monitoring prospective fatigue induced limb asymmetries in HSS, using the Limb Symmetry Index (LSI), might give further insights into the reasons of the increasing HSI incidence over the course of a soccer match.

The aim of this study was therefore to assess HSS of both legs on seven occasions during a simulated soccer match to account for a possible increase in hamstring muscle fatigue (= significant decrease in HSS) in the dominant and the non-dominant leg; this also involves the role of a deterioration of the LSI due to acute hamstring muscle fatigue over the course of a simulated soccer match. Based on specific studies on HSI in real soccer [1, 3, 21], we therefore expected that HSS inversely depicts the HSI incidence rates during real soccer matches; i.e. HSS decreases over the course of each half and HSS at the beginning of the first and second half displays similar values due to the comparably low HSI incidence rate at these occasions. We furthermore expected that the LSI also deteriorates over the course of each half with no differences between the beginning of the first and the second half. Repeated measures ANOVA of both HSS and LSI were used to inferentially analyze potential changes of these respective parameters during a simulated soccer match. These information can help to understand when hamstring muscle fatigue is present during a soccer match and if there is an acute deterioration of the LSI due to hamstring muscle fatigue, which could contribute to the HSI incidence pattern of real soccer matches.

## Methods

### Subjects

10 active soccer players (Bavarian State Division) (age: 21.4 ± 2.9 years, mass: 68.7 ± 5.5 kg, height 1.75 ± 0.06 m) took part in the study. The study was approved by the Technical University of Munich’s Ethics Committee (#2023-415-S-SB) and was conducted between December 11^th^ 2023 and January 24^th^ 2024 according to the Declaration of Helsinki. Informed written consent was obtained from all participants, who were free from acute or chronic injury at the time of data acquisition and had no severe injuries–i.e. they did not miss training or match for more than three consecutive days due to injury–in the lower limbs in the previous 12 months.

### Procedure

The participants carried out the Loughborough Intermittent Shuttle Test (LIST) [54] to simulate the demands of a real soccer match, as used in previous studies [32, 34, 55]. Three to five days prior to the actual testing, participants underwent a familiarization session. In this session, they also completed the Yo-Yo Intermittent Recovery Test I [56] to define their individual maximum aerobic speed (MAS), according to which the LIST was carried out. Furthermore, participants were familiarized with the optimized 90:20 IPCT to assess HSS, and additionally carried out one 15-minute block of the LIST to get accustomed to the procedure and timing intervals of the LIST. Before both the familiarization session and the data acquisition session in the context of the LIST, the participants received a warm-up on a cycle ergometer, on which they rode for 5 minutes at 100 W and 2 minutes at 150 W at 60–70 rpm. In addition, they carried out a more specific standardized warm-up before the actual acquisition of HSS before the LIST, which consisted of:

- 5 handwalks, repeated twice with 60 s of break in between
- Standing scale with 5 repetitions per leg, repeated twice with 60 s of break in between
- 12 repetitions of both-legged bridging
- 6 repetitions of single-legged bridging for each leg
- 12 squats
- 6 counter movement jumps, repeated twice with 60 s of break in between
- 30 s of shoulder bridge with elevated feet, repeated twice with 30 s of break in between
- Single leg shoulder bridge with elevated feet with 12 air time leg changes
- one 20 m sprint at 70, 80, 90% of subjective maximal speed, each, with 30 s of break in between

**Fig 1:**
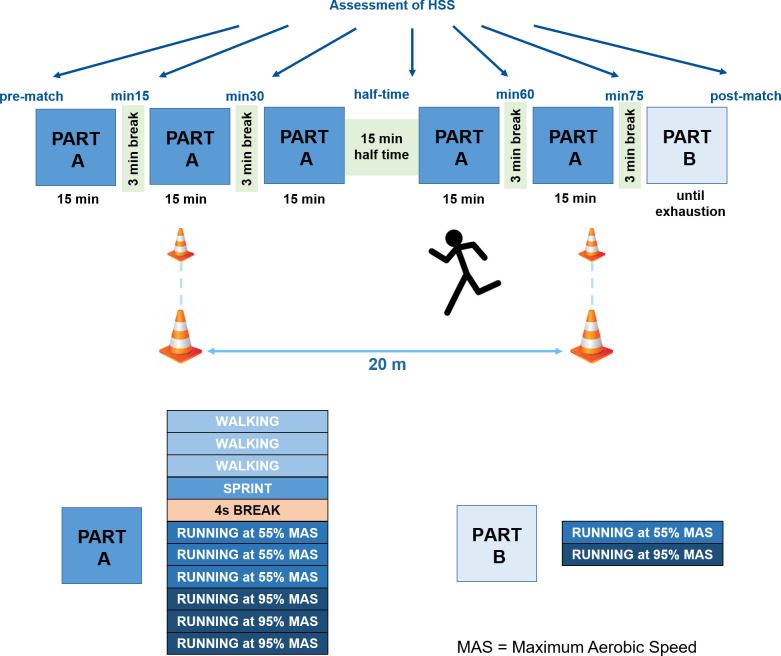
Procedure of the Loughborough Intermittent Shuttle Test.

The LIST itself comprised five 15-minute blocks, which consisted of 20 m lanes of repeated walking at 1.5 m/ s, maximum sprinting, 4 s break, and running at 55% and 95% of the subjects’ individual MAS, respectively (Fig 1). At the end of each 20 m sprint, the participants had to hit a buzzer to activate the 4 s break. The 6^th^ block of the LIST only comprised running at alternating speeds of 55% and 95% of MAS with no break in between until full exhaustion; i.e. participants failed to stay within the individual time limit or voluntarily stopped due to fatigue. There were 3 minutes of break between each block, or 15 minutes of half-time break after the third block, respectively (Fig 1). Isometric HSS via force plate was assessed right before the beginning of each half, during the 3-minute breaks within the LIST and post-LIST. This study was part of a larger project, why we also acquired five repetitions of both eccentric and concentric isokinetic HSS at 180°/ s for each leg pre- and post-LIST and during the half-time break [57] (data will be part of a separate paper). As the assessment of isometric HSS was preceeded by the assessment of isokinetic HSS, the isometric post-match tests, which are part of this study, took place about 10 minutes post-LIST.

Participants received beep-sounds via a customized Matlab script (Version R2020b, MathWorks Inc., Natick, Massachusetts, USA) (available on request from the corresponting author) to control the timing of the 20 m intervals and break durations. Additionally, the examiner, who gave maximal verbal encouragement during both the LIST and the assessment of HSS, also moderated the LIST-procedure to ensure its correct execution. To avoid unilateral fatigue in one leg, participants were asked to alternate their cutting leg at each change of direction movement of the 20 m lanes. The participants wore their regular trainers and carried out the LIST on an outdoor hard surface right next to the lab where the assessment of HSS took place. Therefore, transition times from the LIST to the assessments of HSS could be kept below one minute, why it was feasable to measure HSS of both legs during the 3-minute breaks of the LIST. Drinking ad libitum was allowed during the breaks or the lanes at walking speed, if desired. Eating was also allowed during the 15-minute half-time break.

### Assessment of hamstring strength

**Fig 2:**
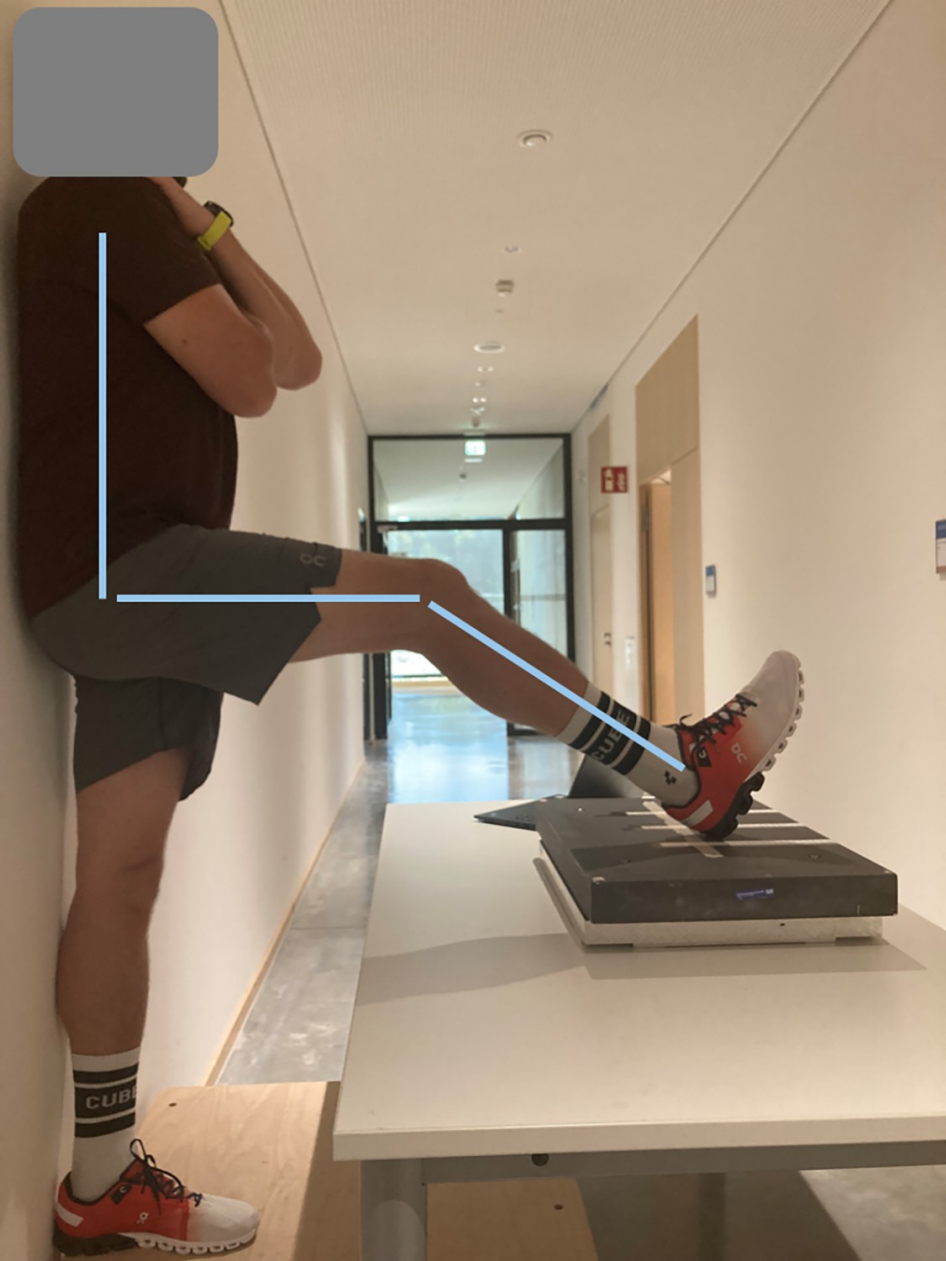
Set-up of the optimized 90:20 Isometric Posterior Chain Test.

The subjects’ HSS was assessed via the optimized 90:20 IPCT, as this procedure showed good to excellent reliability (ICC: 0.94; 95%CI: 0.87–0.98) and very good agreement with gold standard (r = 0.85) in a previous study [24]. To do so, subjects stood with their back against the wall and placed the heel of the leg to be tested onto a force plate (FP4060-10-TM-2000, Bertec, Columbus, Ohio, USA), which was fixed on a height-adjustable table (Sympas STS JCHT35K28A, Assmann Büromöbel GmbH & Co., Melle, GER). With the help of a manual goniometer (Model 01135, Lafayette Instruments Co., Sagamore, Indiana, USA), the participants’ hip and knee flexion angles were set to 90° and 20° degrees, respectively. Participants were then instructed to apply maximum vertical force onto the force plate and pull the force place towards them simultaneously. This was repeated twice for each leg with 30 s break between the trials and an alteration of the leg after each trial. To ensure the same heel positioning during the seven assessments of HSS throughout the LIST, we marked the position of the heel with a piece of tape (Fig 2). We calculated the force perpendicular to the shank using the vertical and posterior horizontal forces, as described in a previous study [24]. This force was then multiplied by the subjects’ shank length, which had been measured to the nearest 0.5 cm with a tape measure beforehand. This calculation provided the applied moment [Nm] onto force plate, serving as a parameter for HSS. The starting leg was randomized and alternated at each occasion of HSS assessment. For statistical analysis, we included the best trial (= largest value of HSS) of each leg at each of the seven occasions during the LIST.

To calculate the limb symmetry index (LSI), we devided the HSS of the weaker the by the HSS of the stronger leg for each of the seven occasions. If the dominant leg was the stronger one, we assigned a positive sign to the LSI; vice versa, if the non-dominant leg was the stronger one, we assigned a negative sign. Here, the dominant leg was defined as the leg the subjects would favor for kicking a ball.

All data were recorded at 1000 Hz with a 16-bit A/D converter (NI USB-6218, National Instruments, Austin, Texas, USA) and the software ProEMG 2.1.0.1 (Prophysics AG, Kloten, CH). Data were smoothed with a 10 Hz zero-lag 4^th^ order Butterworth low-pass filter.

### Statistical analysis

We first calculated the mean and standard deviation of absolute HSS for each leg and measurement. To assess the development of HSS throughout the LIST, we carried out a repeated measures ANOVA for each leg (factor *time*: 7 levels). Additionally, a two-factor (i.e. factor *leg*: 2 levels; factor *time*: 7 levels) repeated measures ANOVA was used to check for differences in HSS between the legs at each of the occasions. A repeated measures ANOVA (factor *time*: 7 levels) was also carried out to see, if there was a change in the LSI over the course of the LIST. The level of significance was set to α = 0.05 and Bonferroni-adjusted for the post-hoc tests in case of a significant ANOVA. No violations against sphericity, homogeneity or normality were present for the ANOVAs. Effect sizes of the post-hoc tests were given as Cohen’s d and regarded as small, medium, and large for values above 0.2, 0.5, and 0.7, respectively [58]. All statistical analyses were computed with JASP 0.16.4 [59].

## Results

Based on the Yo-Yo Intermittend Recovery Test I, which was carried out during the familiarization session, the subjects possesed a MAS of 4.4 ± 0.26 m/ s, according to which the individual times of the 20 m shuttles of the LIST were calculated. During the LIST, the participants covered a total distance of 10.4 ± 0.3 km.

Absolute HSS of the dominant leg was 117 ± 24 Nm for the pre-match values. After 15, 30, 45, 60, and 75 min of the LIST, HSS of 108 ± 20 Nm, 103 ± 18 Nm, 102 ± 24 Nm, 101 ± 14 Nm, and 97 ± 17 Nm, respectively, were measured. Post-match HSS for the dominant leg was 106 ± 20 Nm. The assessment of HSS of the non-dominant leg at the respective occasions resulted in 115 ± 17 Nm, 103 ± 14 Nm, 102 ± 17 Nm, 104 ± 17 Nm, 101 ± 15 Nm, 101 ± 14 Nm, and 105 ± 19 Nm, respectively (processed data and raw data of this study are attached as supplementary files S1 and S2 Raw data).

**Fig 3:**
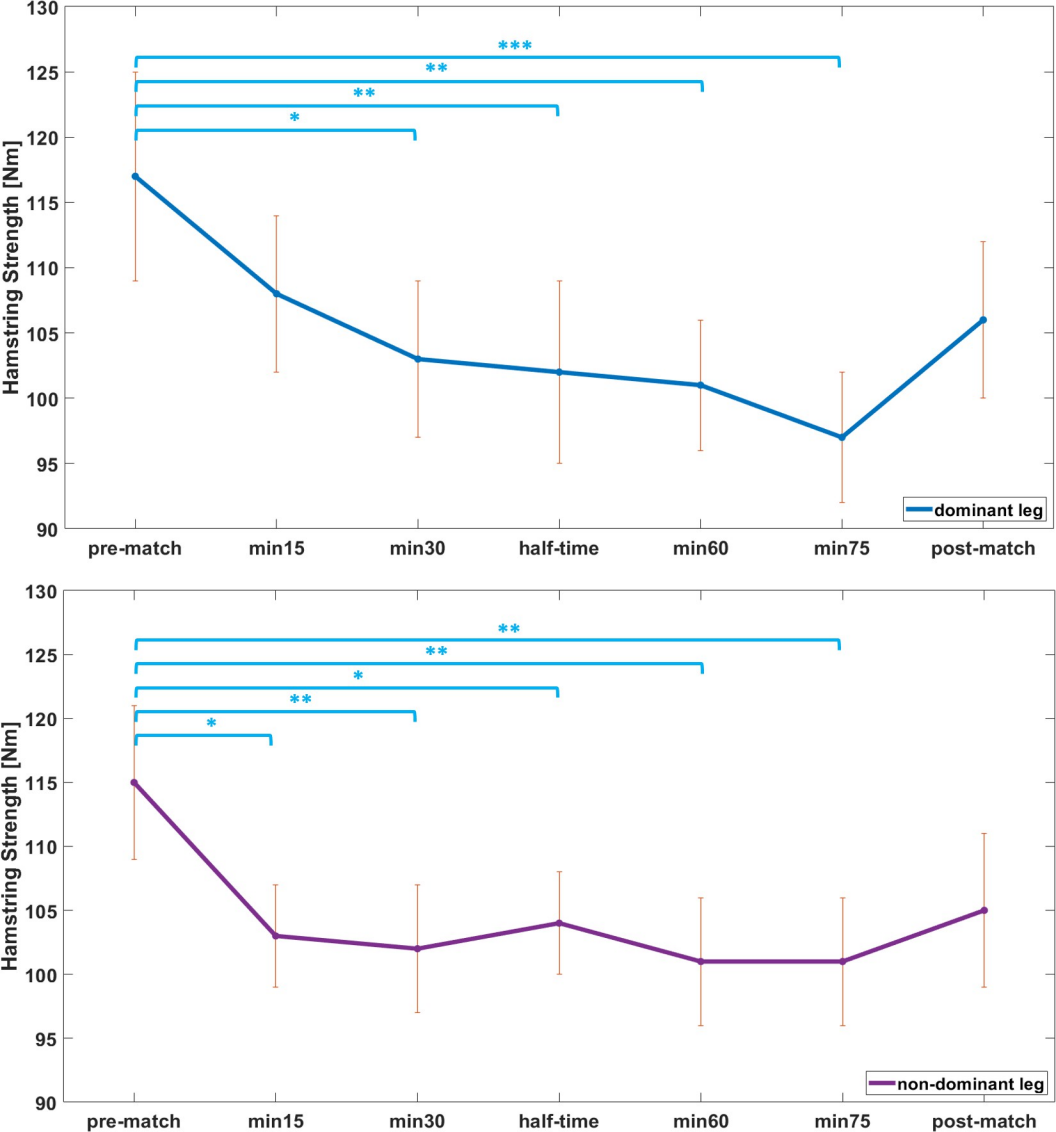
Development of hamstring strength throughout the LIST. Top: dominant leg; Bottom: non-dominant leg. Results are presented as mean ± standard error. *: p < 0.05; **: p < 0.01; ***: p < 0.001.

The repeated measures ANOVA showed a significant effect of match time on HSS for both the dominant (p < 0.001, η^2^ = 0.376) and the non-dominant leg (p < 0.001, η^2^ = 0.332). For the dominant leg, post-hoc tests revealed a significant decrease of HSS compared to pre-match values of 11% at min30 (p = 0.014, d = 0.69), 12% at half-time (p = 0.005, d = 0.75), 12% at min60 (p = 0.003, d = 0.79), and 15% at min75 (p < 0.001, d = 0.96). No further significant differences in HSS between any of the occasions were found for the dominant leg (i.e. p > 0.05). For the non-dominant leg, HSS significantly decreased from pre-match to min15 by 9% (p = 0.017, d = 0.70), min30 by 10% (p = 0.004, d = 0.78), half-time by 10% (p = 0.019, d = 0.69), min60 by 11% (p = 0.002, d = 0.84), and min75 by 11% (p = 0.002, d = 0.84) (Fig 3). Between the pre- and post-match values of the non-dominant leg, a tendency in the decrease of HSS of 8% (p = 0.090, d = 0.58) was detected. Alike the dominant leg, the non-dominant leg showed no further statistically significant differences in HSS between other occasions throughout the LIST.

**Fig 4:**
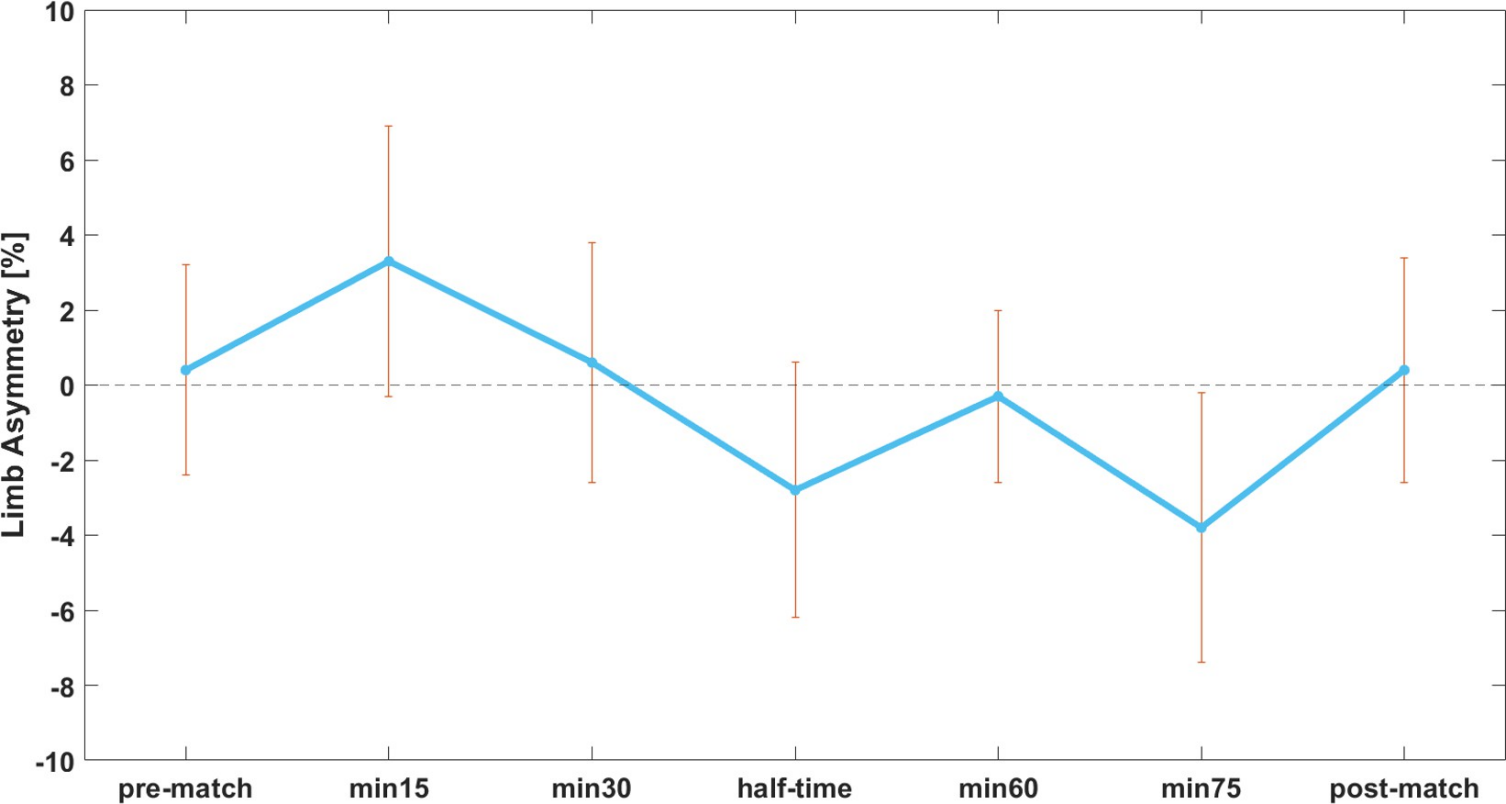
Development of the limb symmetry index (LSI) over the course of the LIST. Results are presented as mean ± standard error. Positive values on y-axis indicate that the dominant leg was stronger; negative values indicate that the non-dominant leg was stronger.

Furthermore, the two-factor repeated measures ANOVA showed no significant differences between the dominant and non-dominant leg at any of the occasions during the LIST (p = 0.987). Regarding the LSI, the repeated measures ANOVA did not detect a significant change throughout the LIST (p = 0.279) with LSI values of 0.4 ± 8.8% at pre-match, 3.3 ± 11.4% at min15, 0.6 ± 10.2% at min30, -2.8 ± 10.9% at half-time, -0.3 ± 7.3% at min60, -3.8 ± 11.3% at min75, and 0.4 ± 9.6% at the post-match measurement, respectively (Fig 4).

A summary of the development of the HSS and LSI data over the seven occasions of the LIST can be found in Table 1.

**Table 1:** Summary of HSS and LSI during the LIST.

	pre-match	min15	min30	half-time	min60	min75	post-match
**HSS dl [Nm]**	117±24	108±20	103±18^a^*	102±24^a^**	101±14^a^**	97±17^a^***	106±20
**HSS n-dl [Nm]**	115±17	103±14^a^*	102±17^a^**	104±17^a^*	101±15^a^**	101±14^a^**	105±19
**LSI [%]**	0.4±8.8	3.3±11.4	0.6±10.2	-2.8±10.9	-0.3±7.3	-3.8±11.3	0.4±9.6

HSS: Hamstring strength; dl: dominant leg; n-dl: non-dominant leg; LSI: Limb Symmetry Index (positive values indicate that the dominant leg was stronger; negative values indicate that the non-dominant leg was stronger); data presented as mean ± standard deviation; ^a^: significantly different to pre-match values with

*: p < 0.05

**: p < 0.01

***: p < 0.001.

For a better understanding of the statistical power of our analyses, we furthermore calculated post-hoc tests according to Guo et al. [60] via using G*Power Statistics (Version 3.1.9.7) [61] and can confirm sufficient power for our sample size for both HSS (dominant leg: power: 0.99, partial η^2^: 0.376, correlation among repeated measures: 0.84; non-dominant leg: power: 0.99, partial η^2^: 0.332, correlation among repeated measures: 0.82) and LSI (power: 0.92, partial η^2^: 0.125, correlation among repeated measures: 0.53).

## Discussion

The aim of this study was to track the development of HSS during a simulated soccer match. This also included the moment when hamstring fatigue first set in and whether there was a recovery of HSS to baseline (= pre-match) levels at the beginning of the second half. The difference in HSS between the dominant and the non-dominant leg, and the change in limb asymmetries throughout a simulated soccer match were also objects of analysis. Via these parameters, conclusions should be drawn if the development of HSS depicts HSI incidences during real soccer matches [1, 3, 21].

Our results show that HSS is significantly decreased after 30 minutes of LIST for the dominant leg, and 15 minutes for the non-dominant leg compared to the respective pre-match values. Bendiksen et al. [62] showed that muscle lactate and creatine phosphate was lowered after already 15 minutes of simulated soccer match, which may explain the fast decrease of HSS in our study. Most studies, which assessed HSS pre-match, during half-time, and post-match, measured the first significant decrease in HSS after 45 minutes of simulated soccer match [40–42, 44, 48, 63], which was the first possible time of detecting hamstring fatigue. Only Robineau et al. and Salter et al. [42, 43] detected a significant loss of HSS compared to the pre-match values only at the post-match measurements. Also among the studies, which assessed HSS on seven or eight occasions over the course of a simulated soccer match, most detected the first significant decrease in HSS only after 45 minutes [44, 45, 48, 49], which was later than in our study (after 15 and 30 minutes, respectively). Wilmes et al. [46] reported a significant decrease of HSS over the course of each half and between the first and second half without further inferential analysis of HSS within a simulated soccer match. The development of HSS over the course of their simulated soccer match, however, strongly replicated our data. They furthermore also measured the strongest decrease of HSS between any two occasions during a simulated soccer match from pre-match to min15, like in our study. Also in accordance with our study, Marshall et al. [44] found the first significant decrease in explosive HSS after already 15 minutes of simulated soccer match.

In terms of the moment of lowest HSS within the LIST, the dominant leg showed its lowest values compared to pre-match after 75 minutes (-15%) with a strong effect size of d = 0.96. For the non-dominant leg, the lowest values were found at both min60 and min75 (-11%, each) with an equally strong effect size of d = 0.84, each. Krustrup et al. [64] found muscle glycogen, creatine phosphate and adenosine triphosphate significantly decreased after a real soccer match compared to pre-match, and after intense bouts of exercise in the second half compared to the first half. This decrease in energy supply may impair HSS during the later stages of the LIST, as the hamstring muscles might not be able to withstand the experienced load–mostly prevalent during high speed running or sprinting, respectively, anymore. It may therefore also contribute to the increased HSI rate during the later stages of the match [1, 3, 21, 23].

In our study, we further observed that post-match HSS did not significantly differ from pre-match HSS, although there was a tendency for the non-dominant leg (p = 0.090; d = 0.58; -8%). In terms of pre-post-match comparisons of HSS, most studies found a significant decrease between these two occasions with a decrease in isometric, concentric and eccentric HSS ranging from -8% to -31% [31, 36–38, 42, 46], -4% to -17% [31, 41, 42], and -5% to -24% [31–33, 35, 37, 39–41, 45, 63], respectively. In contrast to this, other studies–like ours–, did not detect a significant decrease in eccentric [43] or concentric HSS [32] in the pre- vs. post-match comparison. It, however, has to be mentioned that the lowest values in HSS in our study were not present in the post-match measurement but after min60 and min75 of the LIST, respectively. Marshall et al. [44] showed that central motor fatigue is responsible for the decline in HSS during a simulated soccer match. As central motor fatigue recovers faster than peripheral motor fatigue [65], a fast restorage of the capacity of the central nervous system can be the reason for the (non-significant) increase in HSS from min75 to the post-match measurements in our match–this might be well possible, as the post-match measurements of isometric HSS only took place 10 minutes post-match. However, if the recovery of the central motor capacity is the sole reason for a recovery of HSS, one would expect the same increase of HSS at the end of the half-time break, which we could not detect in our study; instead, post-match HSS was even higher than HSS at the end of the half-time break, although there was equal recovery time before these two assessments of HSS. Due to a lack of literature on that matter, we speculate that the rise in HSS from min75 to the post-match measurements might also be due to increased motivation or happy hormones after the successful accomplishment of the LIST. Furthermore, it may be the case that part B of the LIST imposes lower muscle fatiguing demands on the players (i.e. only running at 55% and 95% of MAS, with no sprinting as in part A) than part A. Nutarelli et al. [30] even measured a significant increase in eccentric HSS after a real soccer match in male players. Also Greig [49] observed an increase in concentric HSS–yet not significant–from min75 to post-match. The fact that he carried out a soccer specific treadmill sprint protocol with 6 equal 15-minute blocks makes a suspected higher motivation during the very last measurement of HSS at least *one* possible additional explanation for the rise in HSS from min75 to post-match. This increase in HSS during the last (= post-match) measurement due to a potential increase in motivation could be of great importance for future studies evaluating hamstring fatigue in a pre-post-match setting, as the largest amount of fatigue does not necessarily have to be visible in the post-match measurements but before *within* the match.

In terms of limb asymmetries, our study did neither find significant differences in HSS between the dominant and non-dominant leg for either occasion, nor a significant deterioration (increase of the absolute value) of the LSI throughout the LIST. This is in accordance with Delextrat et al. [35] and Jones et al. [40], who also did not find significant differences in HSS at any of the occasion between the dominant and non-dominant leg during a simulated soccer match. Our results therefore indicate that the LSI within a soccer match is not significantly influenced by acute hamstring fatigue and is therefore an unlikely contributor to the increase in HSI risk over the course of each half in real soccer [1, 3, 21].

The similarly low HSI incidence at the beginning of both halves [1, 3, 21] was not replicated by the simple loss of HSS during our simulated soccer match, as there was no significant recovery of HSS to pre-match values after half-time in our study, which is well in line with existing literature [40–49, 63]. Instead of this HSI pattern observed by Woods et al. [21], Raya-González et al. [3] and Ekstrand et al. [1], the decline in HSS over the course of the LIST in our study follows the observation of Cloke et al. [23] who registered an increasing incidence of muscle injuries in general throughout youth soccer matches with a plateau after the first quarter of the match. Additionally, any difference in HSS between limbs or a deterioration of the LSI throughout our simulated match can neither be attributed to the HSI incidence pattern observed in real soccer matches [1, 3, 21]. Different supplementation strategies of nutritiants during half-time have an influence on the performance [66, 67]–also in terms of strength–and may therefore be responsible for a reduced HSI rate at the beginning of the second half during real soccer matches. Furthermore, some authors suggest that a re-warm-up before the beginning of the second half lowers the risk of HSI compared to a merely passive half-time [45, 48, 49, 67]. Therefore, both nutrition and the way the half-time break is spent may have a significant influence on both the decline and recovery of HSS and eventually the HSI risk during the second half of a soccer match.

An increased HSI risk at the end of each half [1, 3, 21] or after the second quarter of a match [23] would suspect that the match pace becomes faster in these periods as high speed running or sprinting impose the highest load on the hamstring muscles and would therefore increase the risk of HSI [68]. However, none of the studies on sprinting or high-speed running in soccer indicate that the last 15 minutes of each half are of higher intensity than the other periods [69–74]. Some studies even detected a decrease of high intensity periods during the last 15 minutes of each half [70–72]. Reduced HSS over the course of a soccer match, as observed in our study, might therefore be the cause for a slower game during the later stages of the match/ each half but–based on our results–not the sole reason for an increased HSI risk as observed in the pattern by Woods et al., Raya-González et al., and Ekstrand et al. [1, 3, 21].

Additionally, insufficient recovery time between intense bouts of high-speed running or sprinting further contributes to the HSI risk in real soccer matches [20]. On that note, Bradley et al. [70, 71] observed a significantly longer recovery time after high-intensity running bouts in the last 15 minutes of the match compared to the first 15 minutes. Furthermore, the work:rest ratio during the most intense 5 minutes in a soccer match is increased from 1:12 to 1:2 [75]. These 5 minutes of most intense sprint activity were observed to be followed by a 5-minute period of less than average sprint intensity, where the players need to recover [69–72, 74, 75]. The required recovery periods, however, can often not be taken during a real match setting, as players have to react accordingly to the match situation and carry out accumulated sprints or high intensity runs although being fatigued by previous intense bouts.

An additional factor that may explain the lower HSI incidence during the early stages of the second half [1, 3, 21] is the match appearance of substitution players, who mostly enter the match in the second half in a rested state. These stubstitutes, however, may also contribute to the higher incidences during the later stages of the second half [1, 3, 21, 23] due to a distinct, less energy saving, match participation “strategy”. Waldron and Highton [76] point out that substitution players carry out a “one bout, all out” strategy, which is characterized by a faster fatigue and more sprint activities than non-substitutes as they do not have to conserve their energy for a full 90-minute match. On that note, Bradley and Noakes [69] also found 15% more high-intensity running activities in substitution appearances compared to full match appearances of the same player. Our results show significant fatigue of the hamstring muscles after only 15 (non-dominant leg) or 30 minutes (dominant leg) of match time. Substitution players, who compete in a match for only 15 or 30 minutes, respectively, may therefore be exposed to the same risk of suffering HSI like players who compete for a longer period, if a significant decrease in HSS can be made accountable for HSI during soccer matches.

To sum up, the thigh injury pattern during real soccer matches as observed by Cloke et al. [23] can well be explained by the bare loss in HSS over the course of a simulated soccer match, as observed in our study. The observations by Woods et al., Raya-González et al., and Ekstrand et al. [1, 3, 21], that the HSI rate increases within each half with the beginning of the second half showing the same low incidences like the beginning of the first half, however, cannot be explained by a bare loss in HSS. Instead, it might be the combination of decreased HSS *and* insufficient recovery time after intense bouts of high intensisty running/ sprinting within a match that eventually leads to this HSI pattern. This assertion, however, needs to be proven in future studies and therefore remains speculative for the moment.

## Limitations

This very study only took isometric HSS of soccer players throughout a simulated soccer match into account. The simulated soccer match, as used in our study, did not involve kicking activities, which expose the hamstring muscles to further load and may therefore feature an additional–yet less likely than running/ sprinting–potential mechanism leading to HSI [1, 21, 77, 78]. Additionally, equal fatigue in either limb was anticipated by alternating the cutting legs during the change of direction movements in our study. In a real soccer match, however, equal fatigue in both legs cannot be taken for granted due to the variability of match situations and playing positions. Therefore, the difference in fatigue between the two legs and the development of the LSI might be different during a real soccer match compared to a simulated one. Furthermore, the maximum sprint distance in the LIST was 20 m and the recovery times were standardized. During a real match, sprint distances well over 20 m are possible, and players might not have sufficient recovery time after intense bouts of activity, which can increase the risk of HSI due to a possible temporarily more pronounced decrease in HSS. As this study was part of a larger project, the assessment of isometric HSS was preceeded by the assessment of isokinetic HSS for the pre-match, half-time and post-match measurements, which might have impaired isometric HSS for these occasions. This influence, however, seems to be very low as the development of isokinetic HSS followed a similar pattern [57] like isometric HSS in this study (i.e. no difference in HSS between half-time and post-match for either leg and mode of contraction; decrease of HSS at half-time compared to pre-match for dominant leg concentric and non-dominant leg eccentric).

In terms of the LIST procedure itself, we recommend to replace part B (only running at 55% and 95% of MAS) with part A activities (walking, sprinting, and running at 55% and 95% of MAS), as consistent muscular demand may help simplify the interpretation of HSS data in future studies–especially regarding the rise in HSS from min75 to the post-match measurements. The suspected psychological aspect (i.e. increased motivation) during the post-match assessments of HSS may deserve explicit focus for future research to avoid possible misinterpretation of pre-post-match comparisons.

## Conclusion and outlook

To sum-up, our study shows that HSS is significantly decreased after 15 or 30 minutes, respectively, which means that players, may be exposed to the risk of HSI after these durations, assuming a loss of HSS is a main predictor for HSI. Furthermore, there is no recovery of HSS to pre-match values at the beginning of the second half. A deterioration of limb asymmetries in HSS was not observed in our study and does therefore not seem to contribute to the increased HSI risk over the course of a soccer match. The development of hamstring fatigue (= significant decrease in HSS) during the simulated soccer match in our study does only explain or replicate one of the observations on the incidence rate of HSI in real soccer matches [23], whereas other observations of the temporal HSI pattern [1, 3, 21] may additionally be influenced by metabolic factors, half-time routines or insufficient recovery phases within a real soccer match. Furthermore, it is still not clear, whether the significant decrease in HSS itself or the duration of match participation under fatigue plays a more important role when it comes to HSI or if HSI mainly occur due to insufficient recovery time after intense bouts of running/ sprinting in a real soccer match. Future studies on HSI incidence in real soccer matches may therefore also not only want to register the time of the match when the injury occurs, but additionally the duration the injured player has participated in the respective match until HSI.

## Supporting information

S1 Raw data (XLSX)

S2 Raw data (XLSX)
